# Exploration of Ruthenium(II/III/VI)–Salen Complexes: From Synthesis to Functional Applications

**DOI:** 10.3390/molecules30173494

**Published:** 2025-08-25

**Authors:** Beata Cristóvão, Dariusz Osypiuk, Agata Bartyzel

**Affiliations:** Department of General and Coordination Chemistry and Crystallography, Institute of Chemical Sciences, Faculty of Chemistry, Maria Curie-Skłodowska University in Lublin, Maria Curie-Skłodowska sq. 2, 20-031 Lublin, Poland; dariusz.osypiuk@mail.umcs.pl (D.O.); agata.bartyzel@mail.umcs.pl (A.B.)

**Keywords:** ruthenium complex, salen, Schiff base, *N*,*O*-donor ligand

## Abstract

This review provides a comprehensive overview of recent advances in the synthesis, structural characterization, and applications of Ru(II), Ru(III), and Ru(VI) complexes, which bear tetradentate Schiff bases of salen type. Ruthenium complexes exhibit catalytic, electrochemical, and biological properties, serving as multifunctional platforms that integrate fundamental aspects of coordination chemistry with potential practical applications.

## 1. Introduction

**Figure 1:**
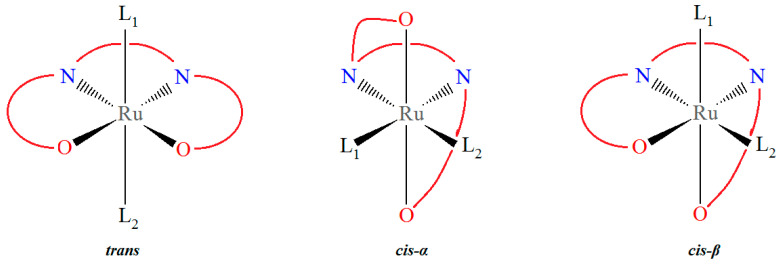
The scheme of the configurations of the ruthenium–salen complexes.

In recent years, ruthenium–salen complexes have been reported as a highly versatile class of compounds, attracting considerable attention due to their diverse potential applications across multiple scientific disciplines. In the context of homogeneous catalysis, these compounds have been the subject of extensive research in the field of oxidation reactions [1,2,3,4,5,6,7,8,9,10,11,12]. The tunable electronic and steric properties of these compounds are conducive to high levels of selectivity, efficiency, and robustness under a range of reaction conditions. Beyond catalysis, their remarkable photophysical features, such as strong absorption, tunable emission, and nonlinear optical responses, have positioned these complexes as promising candidates for the development of advanced optical materials [13,14,15,16,17,18]. Furthermore, within the domain of medicinal chemistry, ruthenium–salen complexes have exhibited remarkable biological activities, including anticancer and antimicrobial properties, along with potential applications as diagnostic and therapeutic agents [18,19,20]. Concurrent with these developments, their rich and reversible redox chemistry has engendered opportunities in electrochemical applications [21], including molecular sensing, electrocatalysis, and the design of novel energy storage systems. The *salen* type Schiff base ligands used for preparation of the Ru–salen compounds are synthesized via the condensation of two equivalents of an *ortho*-hydroxy substituted aromatic aldehyde, most commonly a salicylaldehyde derivative, with a bidentate diamine. The resulting diimine ligand framework is characterized by its ability to chelate metal centers through both nitrogen and oxygen donor atoms, making it highly versatile in coordination chemistry. In the context of an octahedral ruthenium–salen complex, accompanied by two ancillary ligands, three distinct configurations are conceivable. As illustrated in Figure 1, the structures in question are as follows: *trans*, with two apical ancillary ligands, *cis*-*α*, with two equatorial ligands, and *cis-β*, with one apical and one equatorial ligand [22,23]. The N_2_O_2_ donor atoms of the salen ligand and the ruthenium ion in the *trans* complexes are approximately coplanar, while at least one of the two oxygen donor atoms occupies the axial coordination site in the *cis*-*α* or *cis*-*β* complexes. In the case of monodentate ancillary ligands, salen–metal complexes typically adopt a *trans* configuration.

**Figure 2:**
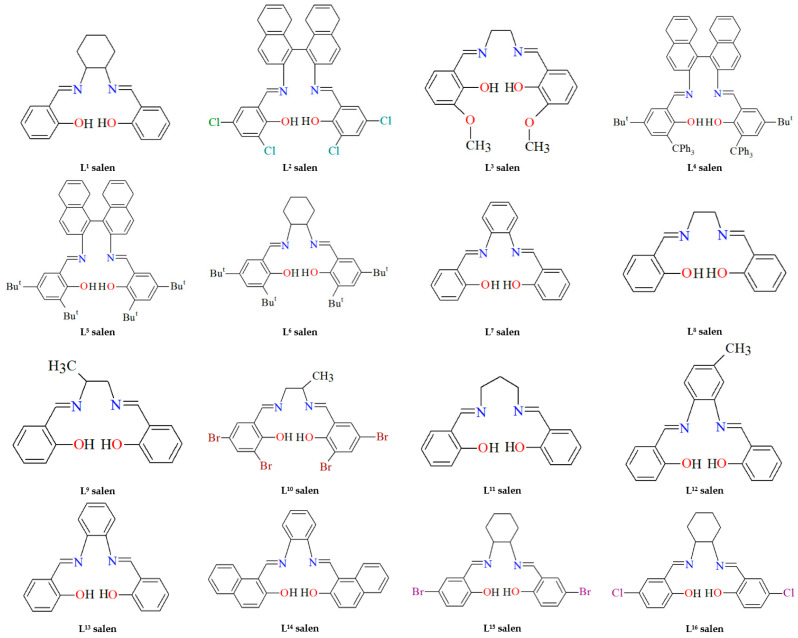
Scheme of tetradentate Schiff base ligands. **L^1^salen** = *N*,*N*′-bis(salicylidene)-*o*-cyclohexyldiamine, **L^2^salen** = *N*,*N*′-bis(3-R^1^-5-R^2^-salicylidene)-(*S*)-(−)-1,1′-binaphthalene-2,2′-diamine; R^1^ = R^2^ = Cl, **L^3^salen** = *N*,*N*′-bis(3-methoxysalicylidene)-1,2-ethylenediamine, **L^4^salen** = *N*,*N*′-bis(3-R^1^-5-R^2^-salicylidene)-(*S*)-(−)-1,1′-binaphthalene-2,2′-diamine; R^1^ = Bu^t^, R^2^ = CPh_3_, **L^5^salen** = *N*,*N*′-bis(3-R^1^-5-R^2^-salicylidene)-(*S*)-(−)-1,1′-binaphthalene-2,2′-diamine; R^1^ = R^2^ = Bu^t^, **L^6^salen** = *N*,*N*′-bis(3-R^1^-5-R^2^-salicylidene)-1,2-cyclohexenediamine dianion; R^1^ = R^2^ = Bu^t^, **L^7^salen** = *N*,*N*′-bis(salicylidene)-1,2-phenyldiamine, **L^8^salen** = *N*,*N*′-bis(salicylidene)-1,2-ethylenediamine, **L^9^salen** = *N*,*N*′-bis(salicylidene)-1,2-(1-methyl)ethylenediamine, **L^10^salen** = *N*,*N*′-bis(3,5-dibromosalicylidene)-1,2-(1-methyl)ethylenediamine, **L^11^salen** = *N*,*N*′-bis(salicylidene)-1,2-propanediamine), **L^12^salen** = *N*,*N*′-bis(salicylidene)-1,2-tolyldiamine, **L^13^salen** = *N*,*N*′-(1,2-phenylene)-bis(salicylideneimine), **L^14^salen** = *N*,*N*′-1,2-phenylene-bis(2-hydroxy-1-naphthylmethyleneimine), **L^15^salen** = *N*,*N*′-bis(5-bromosalicylidene)-*o*-cyclohexyldiamine, **L^16^salen** = *N*,*N*′-bis(5-chlorosalicylidene)-*o*-cyclohexyldiamine.

This review highlights recent developments in the synthesis and functional applications of ruthenium(II), ruthenium(III), and ruthenium(VI) salen complexes (salen ligands, herein designated as L^n^salen, are presented in Figure 2), emphasizing their versatile coordination chemistry, tunable redox properties, and structural diversity. Ru–salen complexes represent promising multifunctional platforms for both fundamental research and practical applications.

## 2. Ruthenium(II/III/VI)–Salen Complexes—Synthesis and Crystal Structures

Mononuclear ruthenium(II/III/VI)–salen complexes **1a**, **2a**, **3a**, **3b**, **4a**, **5a**, **5b**, **6a**, **6b**, **6c**, **7a**, **8a**, **9a**, **9b**, **9c**, **10a**, **11a**, **12a**, **12b**, **13a**, **13b**, **13c**, **13d**, **14a**, **14b**, **15a**, and **15b** (Table 1) were prepared using different synthetic strategies. For instance, complexes **1a**, **9a**, **9c**, **10a**, **11a**, **12a**, **12b**, **13a**, **13b**, **13c**, **13d**, **15a**, and **15b** were synthesized through an indirect route, involving the reaction of ruthenium precursors with the respective tetradentate Schiff base ligands. Complexes **14a** and **14b**, on the other hand, were obtained via a procedure in which the Schiff base ligand was formed in situ during complexation. The heteronuclear ruthenium(III)–salen complexes **16a**, **16b**, **16c**, **17a**, **18a**, **18b**, **19c**, **19b**, **19c**, and **19d** (Table 1) were synthesized using a variety of coordination strategies involving different metal precursors and reaction conditions. For instance, **16a**, **16b**, and **16c** were obtained through the direct reaction of a preformed ruthenium(III)–salen metalloligand with divalent metal perchlorates, such as Mn^2+^, Co^2+^, and Ni^2+^. In the case of **18b**, terbium(III) nitrate was used. The choice of solvent for these reactions varies, with methanol, dichloromethane, ethanol, tetrahydrofuran (THF), acetonitrile, dimethylformamide (DMF), or mixtures commonly employed, depending on the desired reactivity, solubility, and stability of intermediates and final products [24,25,26,27,28,29,30,31,32,33,34,35,36,37,38,39,40,41,42]. In the synthesis of Ru(II/III/VI)–salen complexes, solvent choice is a critical factor that governs the coordination environment and stability of the metal precursor, ligands, and intermediates and directly impacts the catalytic performance of the resulting compounds. It also plays a key role in controlling the oxidation state of ruthenium during complexation. Polar aprotic solvents, such as DMF, DMSO, and ACN, are widely used during the complexation of Ru ions with Schiff bases due to their ability to solvate both the metal precursors and the tetradentate salen ligands. This facilitates efficient complexation and prevents ligand decomposition [1,2,3,4,5,6,7,8,9,10,11,12,13,14,15,16,17,18,19,20,21,22,23,24,25,26,27,28,29,30,31,32,33,34,35,36,37,38,39,40,41,42]. Solvents with low coordinating ability, such as CH_2_Cl_2_ or THF, are often preferred for purification and crystallization processes after synthesis since they allow Ru(II/III) centers to retain high reactivity toward salen coordination, preserve *cis*/*trans* stereochemistry, and prevent the hydrolysis of undesired Ru–O_phen_ or Ru–N_imine_ bonds [24,25,26,27,28,29,30,31,32,33,34,35,36,37,38,39,40,41,42]. In summary, the solvents and synthetic strategies chosen for Ru–salen complexes are not arbitrary, but instead reflect a balance between solubility, stabilization of Ru oxidation states, prevention of unwanted ligand competition, and control of complex nuclearity.

All complexes **1a**–**19d** were comprehensively characterized using a wide range of analytical and spectroscopic techniques. Elemental analysis (C, H, N) was performed for all compounds, and their molecular structures were unambiguously determined by single-crystal X-ray crystallography. Additional spectroscopic studies included IR spectroscopy for all complexes, UV–Vis spectrophotometry for **2a**, **5a**, **5b**, **6a**, **6b**, **6c**, **7a**, **8a**, **9a**–**9c**, **11a**, **14a**, **14b**, and **16a**–**16c**, NMR spectroscopy for **1a**, **4a**, **9a**–**9c**, **10a**, **12a**, **12b**, **14a**, **14b**, **15a**, and **15b**, and EPR spectroscopy for **14a** and **14b**. Electrospray ionization mass spectrometry (ESI-MS) was employed for **3a**, **3b**, **4a**, **5a**, **5b**, and **9a**–**9c**, while cyclic voltammetry was used to investigate the electrochemical properties of **3a**, **3b**, **5a**, **5b**, **12a**, and **12b**. The purity of selected compounds (**1a**, **2a**, **10a**, and **13a**–**13d**) was confirmed by gas chromatography–mass spectrometry (GC–MS). Magnetic susceptibility measurements were carried out for **3a**, **3b**, **5a**, **5b**, **7a**, **8a**, **13a**–**13d**, **16a**–**16c**, **17a**, **18a**, **18b**, and **19a**–**19d**. UV–visible absorption spectra of **6a**, **6b**, and **6c** in CH_2_Cl_2_ at room temperature exhibited bands at ~380 nm and intense absorption below 300 nm, both primarily attributed to intra-ligand charge transfer (ILCT) transitions of the L^2^salen ligand. An additional band near 460 nm was assigned to a metal-to-ligand charge transfer (MLCT) transition. No substantial shifts in these absorption features were observed for complexes **2**–**4**, with complex **2** displaying the longest MLCT wavelength (λ_max_ = 463 nm). In the UV-vis spectra of **9a** and **9c**, broad absorption bands with λ_max_ between 419 and 442 nm were assigned to MLCT transitions. The UV-vis spectrum of **9b** also shows a broad band with λ_max_ at 410 nm, together with a weak low-energy shoulder absorption band at ca. 630−700 nm (log ε~2.69 dm^3^ mol^−1^cm^−1^), which were tentatively assigned to d-d transition. The complexes **6a**–**6c**, **11a**, **14a**, and **14b** were analyzed by density functional theory (DFT) calculations. Single crystals suitable for X-ray crystallographic analysis of the complexes listed in Table 1 were obtained using several approaches. Most frequently, they were grown by recrystallization from the solvent or solvent mixture previously used for synthesis, or from a different solvent. Another employed method involved slow diffusion of a solvent (most often diethyl ether) into a solution of the complexes in an appropriate solvent (commonly methanol or a methanol/dichloromethane mixture). In a few cases, crystals were obtained directly during the synthesis (**1a**) or through slow evaporation of the solvent from the reaction mixture (**4a**, **19a**–**d**).

Structurally, the Ru–salen complexes exhibit a pseudo-octahedral geometry and adopt mainly a *trans* configuration (**1a**, **2a**, **3a**, **3b**, **4a**, **5a**, **5b**, **7a**, **8a**, **10a**, **11a**, **12a**, **12b**, **13a**, **13b**, **13c**, **13d**, **14a**, **14b**), wherein the ruthenium center is coordinated equatorially by two nitrogen and two oxygen donor atoms of the Schiff base ligand, forming a stable N_2_O_2_ coordination environment. The axial positions of the octahedron are typically occupied by either neutral ligands, such as carbon monoxide (CO), nitric oxide (NO), ammonia (NH_3_), water (H_2_O), methanol (CH_3_OH), acetonitrile (CH_3_CN), pyridine (Py), or triphenylphosphine (PPh_3_), or anionic ligands, such as chloride (Cl^−^) or cyanide (CN^−^). The *cis* configuration occurs less frequently (**6a**, **6b**, **6c**, **9a**, **9b**, **9c**, **15a**). The nature of these axial ligands can significantly influence the electronic properties, redox behavior, and catalytic activity of the complexes.

In the case of cationic complexes, counterions such as ClO_4_^−^ (**1a**, **4a**, **16a**, **16b**, **16c**) or PF_6_^−^ (**2a**, **3a**, **3b**, **5a**, **5b**, **11a**) are incorporated into the crystal structure to maintain overall charge neutrality. These anions are typically found in the lattice as non-coordinating species and are often located in the voids or channels of the crystal packing. Although they do not directly participate in coordination to the metal center, their presence can influence the overall crystal packing, intermolecular interactions, and, in some cases, the solubility and stability of the complex. In certain instances, weak hydrogen bonding or electrostatic interactions between the counterions and the coordinated ligands may also contribute to the supramolecular organization of the crystal structure [24,25,26,27,28,29,30,31,32,33,34,35,36,37,38,39,40,41,42].

**Figure 3:**
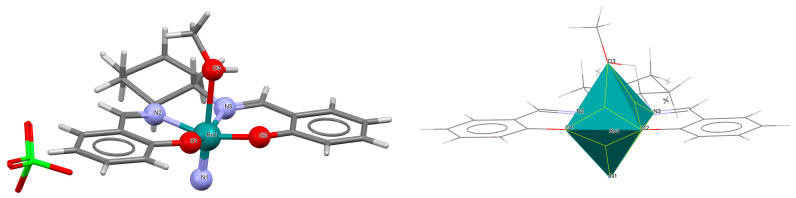
Molecular structure and coordination polyhedron of **1a** IQIBUF [24,25].

A rare high-valent ruthenium–salen complex, [Ru^VI^(N)(L^1^salen)(MeOH)]ClO_4_ **1a** (where L^1^salen = dianion of *N*,*N*′-bis(salicylidene)-*o*-cyclohexyldiamine), was synthesized by reacting [NBu*^n^*_4_][Ru^VI^(N)Cl_4_] with *N*,*N*′-bis(salicylidene)-*o*-cyclohexyldiamine in methanol followed by the addition of ClO_4_^−^ [25]. Compound **1a** (Figure 3, Table 1) exhibits a distorted octahedral geometry, with the L^1^salen ligand coordinated to the Ru(VI) center in the equatorial plane and an axial methanol ligand. The other axial site is occupied by a terminal nitride ion. The distances between Ru(VI) and N_imine_ are 2.030(3) and 2.018(4) Å, while those between Ru(VI) and O_phen_ are 1.977(3) and 1.971(3) Å (Table 1) [24,25].

**Figure 4:**
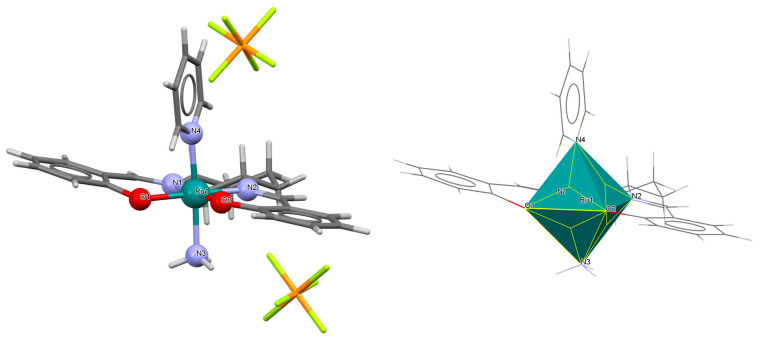
Molecular structure and coordination polyhedron of **2a** EVEHAQ [24,26].

The mononuclear cationic complex [Ru^III^(L^1^salen)(NH_3_)(py)]PF_6_·**2a** was obtained as an oxidation product of the hydroquinone by an electrophilic nitrido species [Ru^IV^N(L^1^salen salen)(MeOH)](PF_6_) (L^1^salen = dianion of *N*,*N*′-bis(salicylidene)-*o*-cyclohexyldiamine) in the presence of pyridine in dichlorometane solution [26]. In the crystal structure of **2a** (Figure 4, Table 1), the Ru(III) ion occupies the N_2_O_2_ site of the Schiff base and exhibits a disordered octahedral environment, where the equatorial plane is composed of two imine nitrogen and two phenolato oxygen atoms of the salen ligand, while two axial positions are occupied by one nitrogen atom of the pyridine and one ammonia nitrogen atom. Bond lengths between Ru(III) and N_imine_ are found to be 1.988(2) and 1.994(3) Å, while those between Ru(III) and O_phen_ measure 2.015(2) and 2.021(2) Å (Table 1) [24,26].

**Figure 5:**
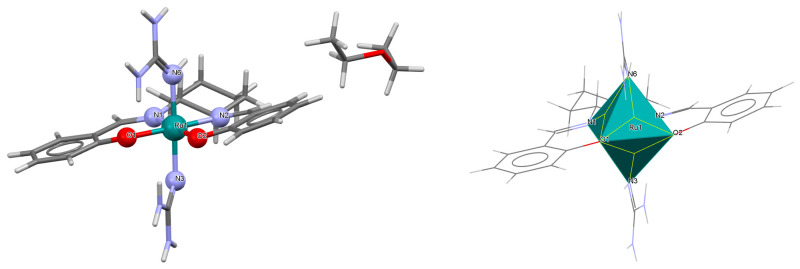
Molecular structure and coordination polyhedron of **3a** LEJLIY [24,27].

**Figure 6:**
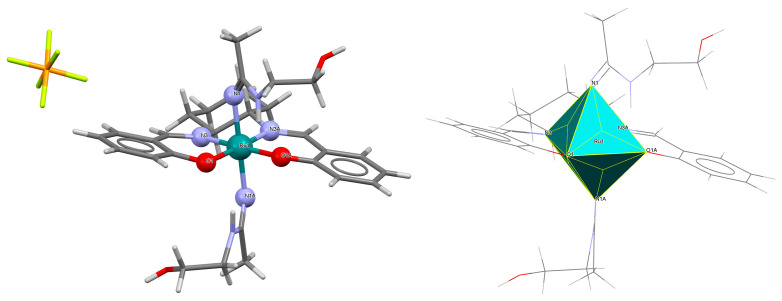
Molecular structure and coordination polyhedron of **3b** LEJLOE [24,27].

The cationic complexes *trans-*{Ru^III^(L^1^salen)[NH=C(NH_2_)_2_]_2_}PF_6_·Et_2_O **3a** and *trans*-{Ru^III^(L^1^salen)[NH=C(NH_2_)(NHC_2_H_4_OH)]_2_}PF_6_ **3b** (where L^1^salen = dianion of *N*,*N*′-bis(salicylidene)-*o*-cyclohexyldiamine) were synthesized by treating the solution of *trans*-[Ru^III^(L^1^salen)(N≡CNH_2_)_2_]PF_6_ in THF at a high temperature with gaseous NH_3_, or by adding ethanolamine to the solution of *trans*-[Ru^III^(L^1^salen)(N≡CCH_3_)_2_]PF_6_ in THF [27]. It is evident (Figure 5 and Figure 6, Table 1) that the ruthenium(III) ion in both mononuclear complexes exhibits a distorted octahedral geometry, with the tetradentate Schiff base situated in the equatorial plane while the axial positions are occupied by two nitrogen atoms from two guanidine **3a** or two amidine **3b** ligands. The distances between Ru(III) and N_imine_ range from 1.981 to 1.990 Å, while those between Ru(III) and O_phen_ lie between 2.023 and 2.032 Å (Table 1) [24,27].

**Figure 7:**
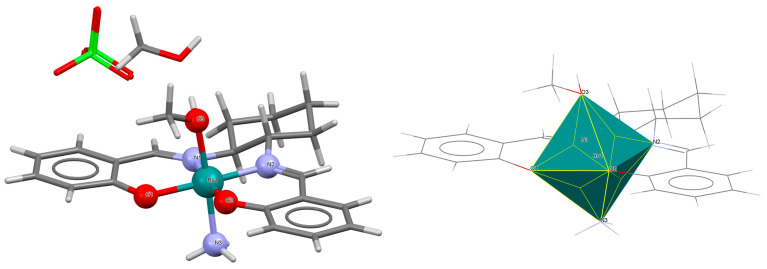
Molecular structure and coordination polyhedron of **4a** NIGDEP [24,28].

Another mononuclear cationic complex [Ru^III^(L^1^salen)(NH_3_)(MeOH)]ClO_4_·MeOH **4a** was isolated as the final product resulting from the simple reduction of a ruthenium(VI) nitride complex [Ru^VI^(L^1^salen)(N)(OH_2_)]^+^ to [Ru^III^(L^1^salen)(NH_3_)(OH_2_)]^+^ [28]. This transformation proceeds via ruthenium(IV) sulfilamido, ruthenium(III) sulfilamine, and ruthenium(IV) amido intermediates upon reaction with L-cysteine in aqueous acidic solution. Kinetic and mechanistic studies in this case suggest that the reaction involves proton-coupled electron transfer processes. In the molecular structure of **4a** (Figure 7, Table 1), the ruthenium(III) is octahedrally coordinated by a tetradentate salen Schiff base ligand in the plane as well as by one ammonia and one methanol molecule in the apical positions. As shown in Table 1, Ru–N_imine_ bond lengths were determined to be 1.988(3) and 1.989(3) Å, and the Ru–O_phen_ distances were found to be 2.015(2) and 2.028(2) Å [24,28].

**Figure 8:**
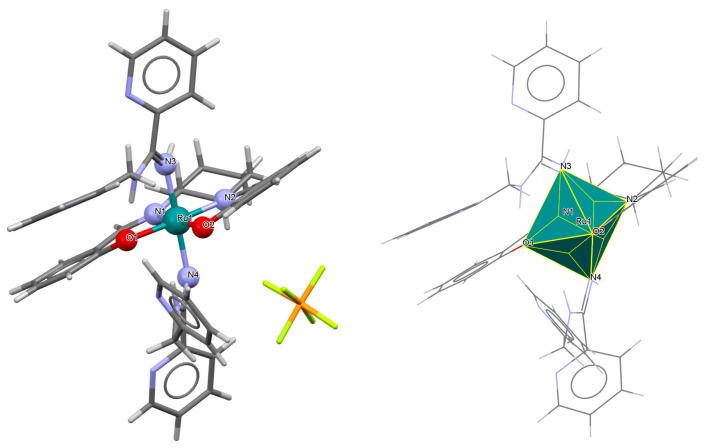
Molecular structure and coordination polyhedron of **5a** QAKJIZ [24,29].

**Figure 9:**
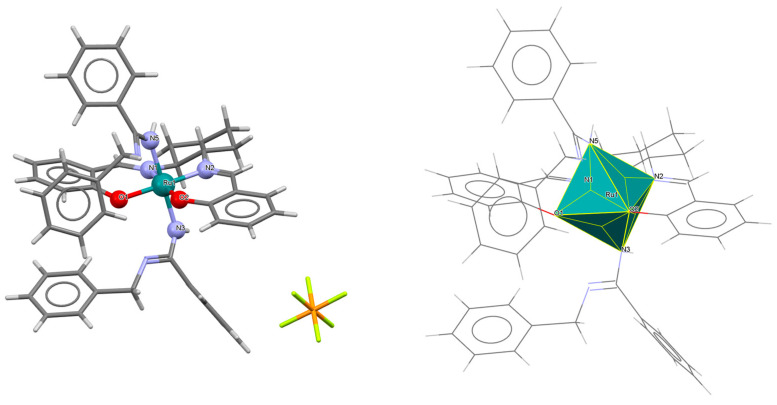
Molecular structure and coordination polyhedron of **5b** QAKJOF [24,29].

The mononuclear paramagnetic complexes [Ru^III^(L^1^salen)(NHC(NHCH_2_Py)Py)_2_]PF_6_ **5a** and [Ru^III^(L^1^salen)(NHC(NHCH_2_Ph)Ph)_2_]PF_6_ **5b** were synthesized during reaction of [Ru^III^(L^1^salen)(H_2_O)_2_](PF_6_) with 2-(aminomethyl)pyridine (Py) or benzylamine (Ph) [29]. Compounds **5a** and **5b** (Figure 8 and Figure 9, Table 1) exhibit a distorted octahedral geometry, with the ruthenium(III) centers coordinated to two oxygen atoms and two nitrogen atoms from the N_2_O_2_ salen ligand in the equatorial plane. The axial positions are occupied by two nitrogen atoms from the amidine ligands. In the crystal structures of **5a** and **5b**, the axial arylamidine ligands were generated in situ through the oxidative coupling of two molecular amines, marking the first reported instance of a direct metal-mediated coupling between amines. This transformation is air-sensitive, as no analogous products were formed under an inert atmosphere, and it is strongly dependent on the nature of the amine substrate. The distances between Ru(III) and N_imine_ range from 1.987 to 2.067 Å, while those between Ru(III) and O_phen_ lie between 2.015 and 2.027 Å (Table 1) [24,29].

**Figure 10:**
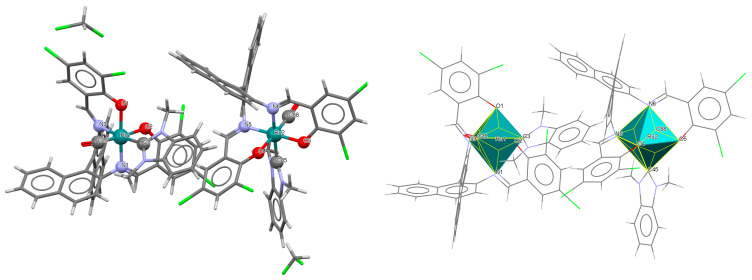
Molecular structure and coordination polyhedra of **6a** ERUQIU [24,30].

**Figure 11:**
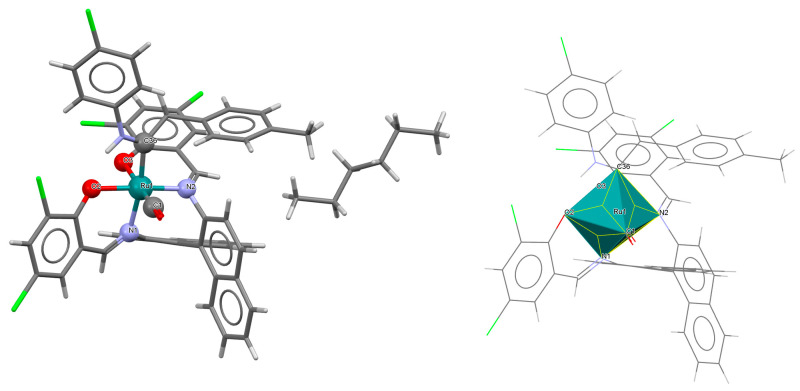
Molecular structure and coordination polyhedron of **6b** ERUQOA [24,30].

**Figure 12:**
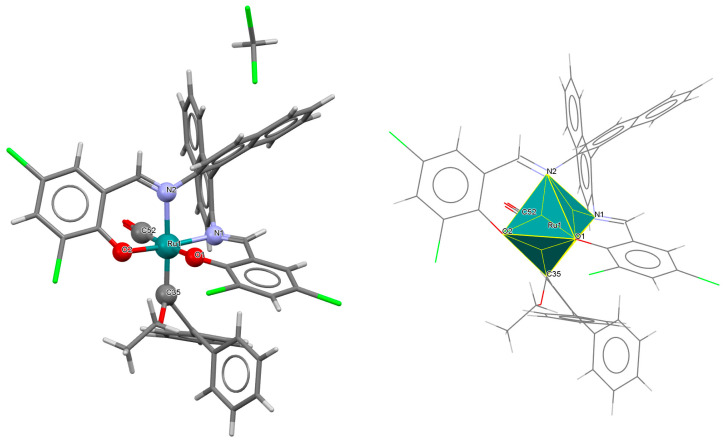
Molecular structure and coordination polyhedron of **6c** ERUQUG [24,30].

The mononuclear neutral complexes *cis*-*β*-[Ru^II^(L^2^salen)(CO)L^2a^]·2CH_2_Cl_2_ **6a**, *cis*-*β*-[Ru^II^(L^2^salen)(CO)L^2b^]·0.5C**_6_**H**_14_ 6b**, and *cis*-*β*-[Ru^II^(L^2^salen)(CO)L^2c^]·CH_2_Cl_2_ **6c** were synthesized by the reaction of *cis*-*β*-[Ru^II^(L^2^salen)(CO)_2_] (L^2^salen = dianion of *N*,*N*′-bis(3-R^1^-5-R^2^-salicylidene)-(*S*)-(−)-1,1′-binaphthalene-2,2′-diamine; R^1^ = R^2^ = Cl) with heteroatom-stabilized carbene ligands, such as 3-dimethyl-1H-benzimidazolium iodide (L^2a^) in tetrahydrofuran, *p*-chloro-aniline and *p*-methyl-phenylacetylene (L^2b^) **6b** in 1,2-dichlorometane, and 1,1-diphenyl-2-propyn-1-ol (L^2c^) in ethanol [30]. The synthesis of the compounds did not require photolytic decarbonylation, i.e., irradiation with a 300 W incandescent lamp. In all complexes (Figure 10, Figure 11 and Figure 12, Table 1), the ruthenium(II) ion adopts an octahedral configuration, with the coordination sphere of the metal center consisting of two nitrogen and two oxygen atoms from the doubly deprotonated tetradenate Schiff base (in a *cis*-salen configuration), as well as two carbon atoms from the carbonyl group and the carbene ligand. The distances between Ru(II) and N_imine_ range from 2.036 to 2.178 Å, while those between Ru(II) and O_phen_ lie between 2.060 and 2.101 Å (Table 1) [24,30].

The donor atoms of the L^2^salen ligand (N or O) adjacent to the carbene C atom contribute to the high stability of **6a**, **6b**, and **6c** both in solution and in solid state [30].

**Figure 13:**
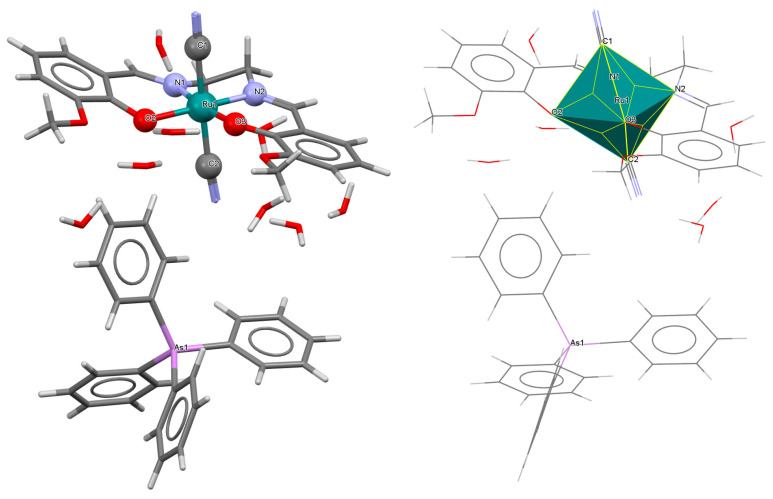
Molecular structure and coordination polyhedron of **7a** GOJKEY [24,31].

The mononuclear anionic complex AsPh_4_[Ru^III^(L^3^salen))(CN)_2_]·8.5H_2_O **7a** (where L^3^salen = dianion of *N*,*N*′-bis(3-methoxysalicylidene)-1,2-ethylenediamine) was obtained during reaction of [Ru^III^(L^3^salen)(PPh_3_)Cl] with KCN and AsPh_4_Cl, respectively [31]. The crystal structure of **7a** (Figure 13, Table 1) is composed of individual *trans*-[Ru^III^(L^3^salen)(CN)_2_]^−^ anions, tetraphenylarsonium counterions, and water molecules. The ruthenium(III) center is located within the N_2_O_2_ donor set of the Schiff base, adopting an octahedral coordination geometry with two imino nitrogen and two phenoxido oxygen atoms in the equatorial plane, and two cyanide ligands occupying the axial positions. The Ru–N_imine_ bond lengths are measured at 1.980(7) and 1.975(7) Å, whereas the Ru–O_phen_ distances are 2.004(4) and 2.025(6) Å (see Table 1) [24,31].

**Figure 14:**
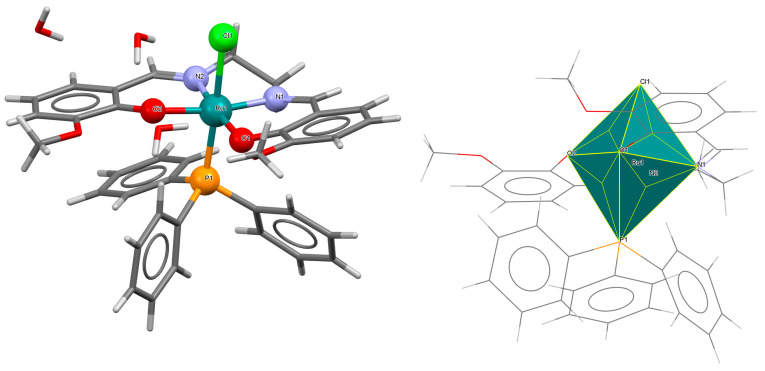
Molecular structure and coordination polyhedron of **8a** HUKLEH [24,32].

The mononuclear neutral complex [Ru^III^(L^3^salen)(PPh_3_)Cl]·3H_2_O **8a** (where PPh_3_ = triphenylphosphine) was synthesized in air by the reaction of [Ru^II^(PPh_3_)_3_Cl_2_] with *N*,*N*′-bis(3-methoxysalicylidene)-1,2-ethylenediamine in methanol, in the presence of triethylamine [32]. As illustrated in Figure 14, the ruthenium(III) ion, which displays an octahedral geometry, is positioned within the N_2_O_2_ compartment of the Schiff base ligand. The equatorial plane is defined by two imino nitrogen atoms and two phenoxido oxygen atoms, while the axial positions are occupied by a chloride ion and the phosphorus atom of the PPh_3_ ligand. Bond lengths between Ru(III) and N_imine_ are found to be 1.958(8) and 1.985(9) Å, while those between Ru(III) and O_phen_ measure 2.008(6) and 2.009(7) Å, as presented in Table 1 [24,32].

**Figure 15:**
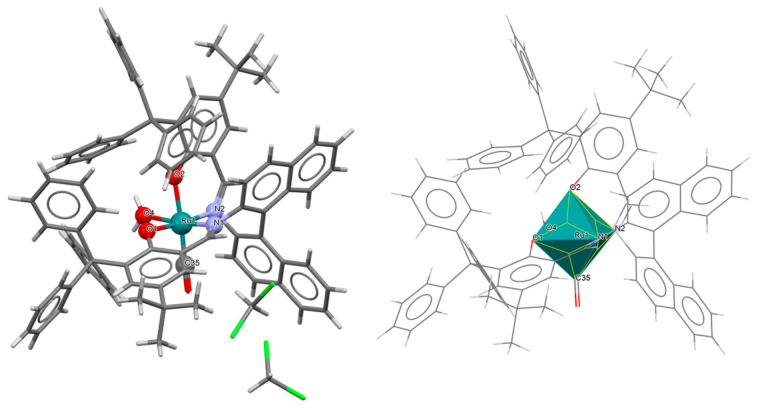
Molecular structure and coordination polyhedron of **9a** KUSVAY [24,33].

**Figure 16:**
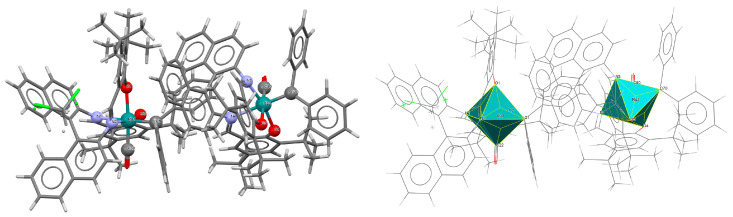
Molecular structure and coordination polyhedra of **9b** KUSVEC [24,33].

**Figure 17:**
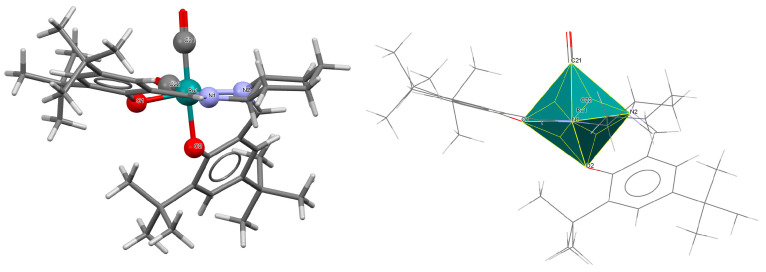
Molecular structure and coordination polyhedron of **9c** KUSXII [24,33].

The unique neutral complexes *cis*-*β*-[Ru^II^(L^4^salen)(H_2_O)(CO)]·2CH_2_Cl_2_ **9a**, *cis*-*β*-[Ru^II^(L^5^salen)(CO)(CPh_2_)]·0.5CH_2_Cl_2_ **9b**, *cis*-*β*-[Ru^II^(L^6^salen)(CO)_2_]·0.25MeOH **9c** (where L^4^salen = dianion of *N*,*N*′-bis(3-R^1^-5-R^2^-salicylidene)-(*S*)-(−)-1,1′-binaphthalene-2,2′-diamine; R^1^ = Bu^t^ R^2^ = CPh_3_, L^5^salen = dianion of *N*,*N*′-bis(3-R^1^-5-R^2^-salicylidene)-(*S*)-(−)-1,1′-binaphthalene-2,2′-diamine; R^1^ = R^2^ = Bu^t^, L^6^salen = dianion of *N*,*N*′-bis(3-R^1^-5-R^2^-salicylidene)-1,2-cyclohexenediamine dianion; R^1^ = R^2^ = Bu^t^) bearing nonplanar N_2_O_2_ salen type ligands were obtained in different ways, i.e., **9a** and **9c** by reaction of Ru_3_(CO)_12_ and the corresponding H_2_L^4^salen/H_2_L^6^salen ligand in 1,2,4-trichlorobenzene under an argon atmosphere in the absence of light; the synthesis of **9b** was carried out by irradiation of a refluxing solution of *cis*-*β*-[Ru^II^(L^5^salen)(CO)_2_] in acetonitrile with an incandescent lamp (300 W) with the objective of removing one of the two coordinated CO groups, and subsequent treatment with a solution of the diazo compound N_2_CPh_2_ dissolved in dichloromethane at room temperature [22,33]. In all crystal structures (Figure 15, Figure 16 and Figure 17, Table 1), the central metal ion is six-coordinated by two nitrogen and two oxygen atoms of the Schiff base in a *cis*-salen configuration and, additionally, the coordination sphere is completed by one carbon atom of the carbonyl group and one aqua oxygen atom (**9a**); a carbon atom of the carbonyl group and a carbon atom of the carbene ligand (**9b**); and two carbon atoms of the carbonyl groups (**9c**). Ru–N_imine_ and Ru–O_phen_ bond lengths are observed within the ranges of 2.026–2.202 Å and 2.060–2.101 Å, respectively.

**Figure 18:**
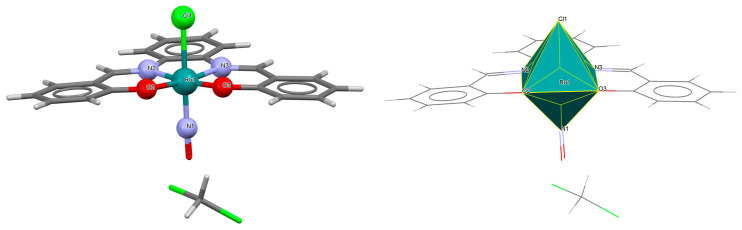
Molecular structure and coordination polyhedron of **10a** LEQBAN [24,34].

A neutral ruthenium(II) nitrosyl complex [Ru^II^(L^7^salen)(NO)Cl]·CH_2_Cl_2_ **10a** (where L^7^salen = dianion of *N*,*N*′-bis(salicylidene)-1,2-phenyldiamine) was obtained at a higher temperature during the reaction of Ru(NO)Cl_3_·xH_2_O, which was dissolved in dimethylformamide (DMF), with *N*,*N′*-bis(salicylidene)-1,2-phenylenediamine in tetrahydrofuran (THF), in the presence of triethylamine [34]. In the crystal structure of **10a** (Figure 18, Table 1), the ruthenium(II) ion is in an octahedral coordination environment containing one tetradentate Schiff base ligand, one chloride ion, and one nitrosyl group. The Ru–N_imine_ bond lengths are reported as 2.020(4) and 2.022(4) Å, whereas the Ru–O_phen_ bonds are 2.023(4) and 2.025(3) Å (Table 1) [24,34].

**Figure 19:**
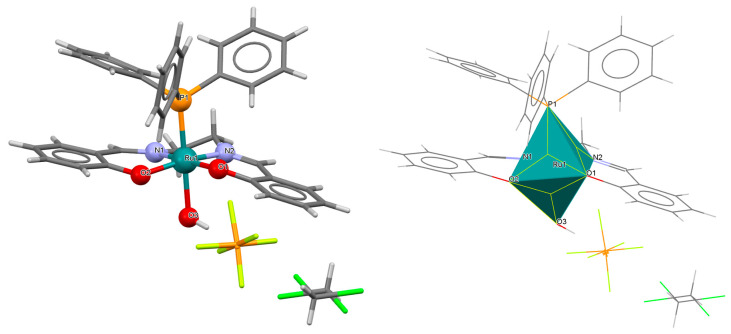
Molecular structure and coordination polyhedron of **11a** MEKGIV [24,35].

The cationic complex [Ru^III^(L^8^salen)(H_2_O)(PPh_3_)]PF_6_·0.5CH_2_Cl_2_ **11a** (where L^8^salen = dianion of *N*,*N*′-bis(salicylidene)-1,2-ethylenediamine, PPh_3_ = triphenylphosphine) was obtained in the reaction of [RuCl_2_(PPh_3_)_3_] with a methanolic solution of *N*,*N*′-bis(salicylidene)-1,2-ethylenediamine [35]. Single crystals of the compound were obtained from dichloromethane. The central ruthenium(III) ion in the [Ru^III^(L^8^salen)(H_2_O)(PPh_3_)]^+^ has a distorted octahedral geometry, with two imino nitrogen atoms and two phenoxy oxygen atoms of the Schiff base forming the equatorial plane in the coordination polyhedron and the axial positions occupied by an aqua oxygen and phosphorus atom of a PPh_3_ moiety (Figure 19, Table 1). The bond distances between the salen donor atoms N1, N2, O1, O2, and Ru(III) are 2.003(6), 1.983(6), 2.008(5), and 2.007(6) Å, respectively (see Table 1) [24,35].

**Figure 20:**
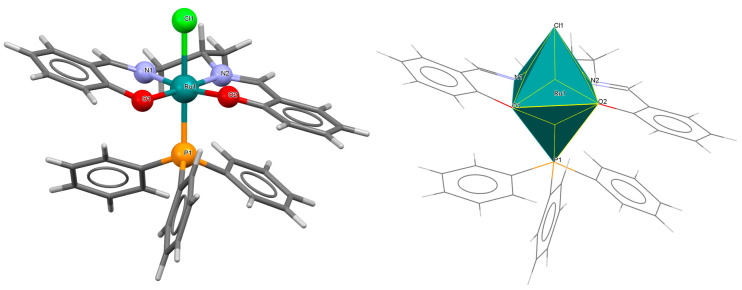
Molecular structure and coordination polyhedron of **12a** YOBXAR [24,36].

**Figure 21:**
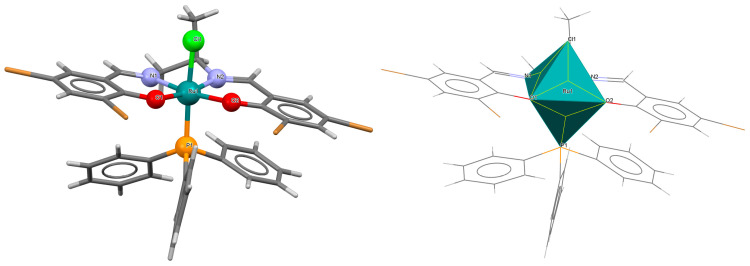
Molecular structure and coordination polyhedron of **12b** YOBXEV [24,36].

The neutral complexes [Ru^III^Cl(L^9^salen)(PPh_3_)] **12a** and [Ru^III^Cl(L^10^salen)(PPh_3_)] **12b** (where L^9^salen = dianion of *N*,*N*′-bis(salicylidene)-1,2-(1-methyl)ethylenediamine, L^10^salen = dianion of *N*,*N*′-bis(3,5-dibromosalicylidene)-1,2-(1-methyl)ethylenediamine, PPh_3_ = triphenylphosphine) were prepared during reaction of [Ru^II^Cl_2_(PPh_3_)_3_] with the respective unsymmetrical tetradentate Schiff bases in tetrahydrofuran solution [36]. During the synthesis of **12a** and **12b**, oxidation of Ru(II) to Ru(III) by air is reported. In the crystals of **12a** and **12b** (Figure 20 and Figure 21, Table 1), N_2_O_2_-donor ligands coordinate to ruthenium(III) in a tetradentate mode. The compounds are octahedral in nature and adopt *trans* configuration. The basal plane of the central atom is formed by the two phenolic oxygen atoms and the two imine nitrogen atoms of the respective Schiff base, while the axial positions are occupied by the chlorine and phosphine atoms. Ru–N_imine_ and Ru–O_phen_ bond lengths are reported within the ranges of 1.966–2.008 Å and 2.008–2.105 Å, respectively (Table 1) [24,36].

**Figure 22:**
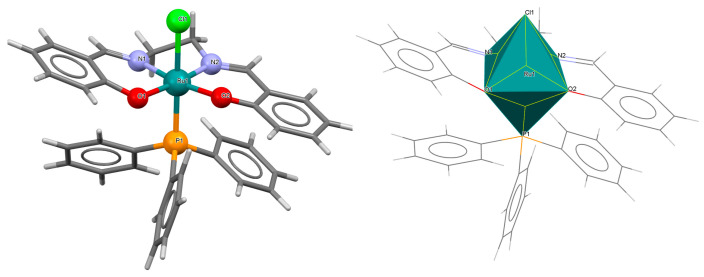
Molecular structure and coordination polyhedron of **13a** ZIBNOQ [24,37].

**Figure 23:**
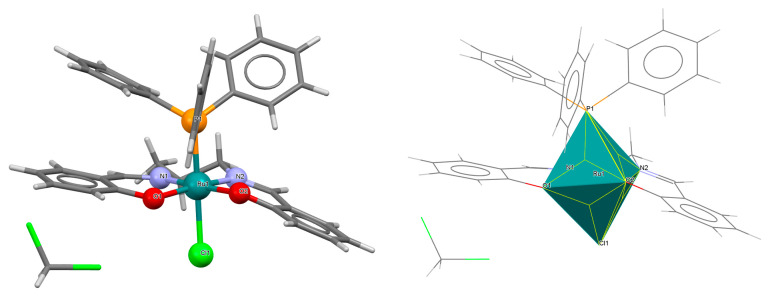
Molecular structure and coordination polyhedron of **13b** ZIBNUW [24,37].

**Figure 24:**
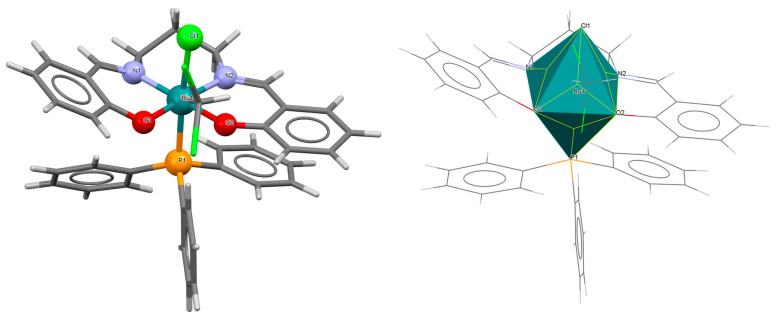
Molecular structure and coordination polyhedron of **13c** ZIBPAE [24,37].

**Figure 25:**
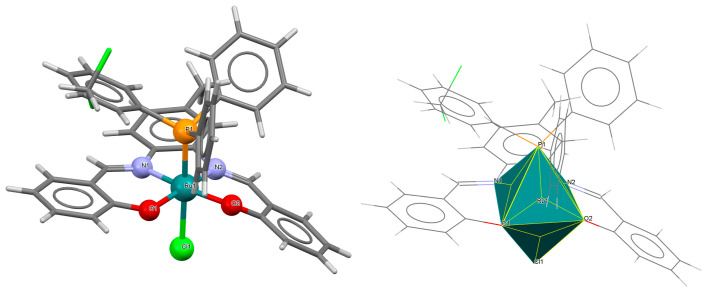
Molecular structure and coordination polyhedron of **13d** ZIBPEI [24,37].

The series of mononuclear neutral complexes [Ru^III^Cl(L^8^salen)(PPh_3_)] **13a**, [Ru^III^Cl(L^9^salen)PPh_3_)]·2CH_2_Cl_2_ **13b**, [Ru^III^Cl(L^11^salen)(PPh_3_)]·CH_2_Cl_2_ **13c** (where L^11^salen = dianion of *N*,*N*′-bis(salicylidene)-1,2-propanediamine), [Ru^III^Cl(L^12^salen)(PPh_3_)]·CH_2_Cl_2_ **13d** (where L^12^salen = dianion of *N*,*N*′-bis(salicylidene)-1,2-tolyldiamine) were obtained during reactions in nitrogen atmosphere [Ru^II^Cl_2_(PPh_3_)_3_] and equal equivalents of the respective Schiff bases in THF/CH_2_Cl_2_ with the presence of a small excess of Et_3_N [37]. In the crystals of all complexes (Figure 22, Figure 23, Figure 24 and Figure 25, Table 1), the central ruthenium(III) ion is in an octahedral coordination environment, containing N_2_O_2_-donor atoms of one Schiff base in the equatorial position and triphenylphosine and chloride ions in the apical positions. The distances between Ru(III) and N_imine_ range from 1.986 to 2.051 Å, while those between Ru(III) and O_phen_ are between 2.004 and 2.022 Å (Table 1) [24,37].

**Figure 26:**
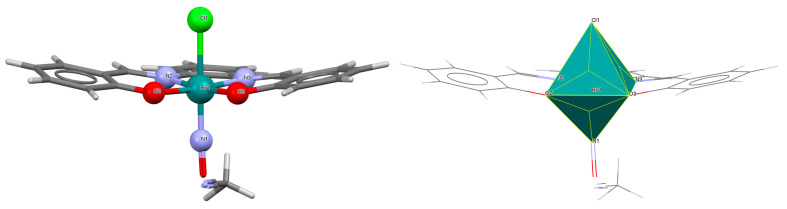
Molecular structure and coordination polyhedron of **14a** CENXAY [24,38].

**Figure 27:**
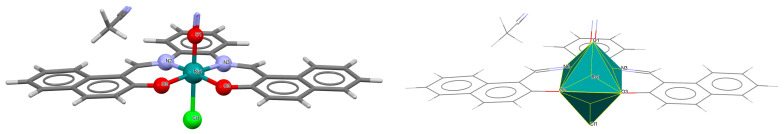
Molecular structure and coordination polyhedron of **14b** CENXIG [24,38].

The other neutral complexes [Ru^III^Cl(L^13^salen)(NO)]·CH_3_CN **14a** and [Ru^III^Cl(L^14^salen)(NO)]·CH_3_CN **14b** (where L^13^salen = dianion of *N*,*N*′-(1,2-phenylene)-bis(salicylideneimine) and L^14^salen = dianion of *N*,*N*′-1,2-phenylene-bis(2-hydroxy-1-naphthylmethyleneimine) were synthesized in a one-pot reaction in ethanol, using salicylaldehyde or 2-hydroxy-1-naphthaldehyde and 1,2-phenylenediamine, along with [Ru(NO)Cl_3_]·xH_2_O and a small amount of Et_3_N [38]. Compounds **14a** and **14b** (Figure 26 and Figure 27, Table 1) exhibit a slightly distorted octahedral coordination environment around the Ru(III) center, where the equatorial plane is defined by the nitrogen and oxygen atoms of the L^13^salen/L^14^salen ligand, and the axial positions are occupied by a NO molecule and a chloride ion. As shown in Table 1, Ru–N_imine_ bond lengths were determined to be in the range of 2.005–2.031Å, and the Ru–O_phen_ distances were found to be 2.027–2.037 Å [24,38].

**Figure 28:**
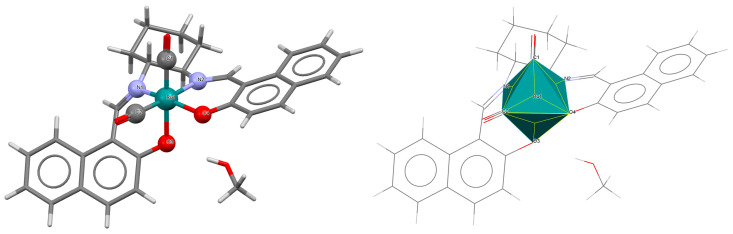
Molecular structure and coordination polyhedron of **15a** ECEZIZ [24,39].

The inert mononuclear complex *cis-β*-[Ru^II^(L^14^salen)(CO)_2_]·MeOH **15a** was synthesized in a one-pot reaction at high temperature between Ru_3_(CO)_12_ and *N*,*N*′-1,2-phenylene-bis(2-hydroxy-1-naphthylmethyleneimine) in the presence of 1,2,4-trichlorobenzene [39]. The axial sites of the octahedrally coordinated Ru(II) center are occupied by a phenolate oxygen from the Schiff base ligand and an oxygen atom from a carbonyl group (Figure 28, Table 1). Bond lengths between Ru(II) and N_imine_ are found to be 2.038(2) and 2.065(2) Å, while those between Ru(II) and O_phen_ measure 2.041(1) and 2.092(1) Å, as presented in Table 1. An alkoxo-bridged dinuclear Ru(II) complex **15b** was obtained as a minor product during **15a** preparation. In its crystal structure (Table 1), each ruthenium(II) center adopts an octahedral coordination geometry, bonded to two CO ligands and a tridentate dibasic ONO-type Schiff base. Notably, the alkoxo oxygen atoms also bridge to the ruthenium(II) ion of an adjacent monomer unit. The Ru–N_imine_ bond lengths are measured at 2.066(3) and 2.075(4) Å, whereas the Ru–O_phen_ distances are in the range of 2.066–2.218 Å (Table 1) [24,39].

**Figure 29:**
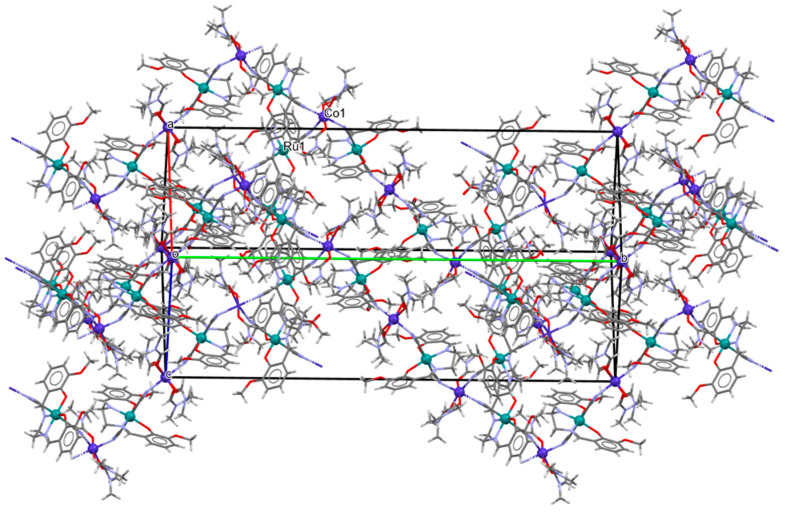
Two-dimensional layered framework of **16a** GOJGUK [24,31].

The ^∞^_2_[{Ru^III^(L^3^salen)(CN)_2_}_4_{M^II^(DMF)_3_}_2_{Mn^II^(DMF)_4_}](ClO_4_)_2_·4DMF **16a**, **16b**, and **16c** (where M^II^ = Mn **16a**, Co **16b**, Ni **16c**) 2D isostructural coordination polymers with ratios of Ru(III):M(II) equal to 4:3 were synthesized through the reaction of AsPh_4_[Ru^III^(L^3^salen))(CN)_2_]·8.5H_2_O with the perchlorate salt of the corresponding divalent metal in a DMF solution [31]. In the crystal structure of **16a** (Figure 29, Table 1), the recurring structural motif features 48-membered metallacycles incorporating two crystallographically distinct ruthenium(III) and manganese(II) centers. One of the hexacoordinated M(II) ions is coordinated by three nitrogen atoms originating from three [Ru^III^(L^3^salen)(CN)_2_]^−^ units and by three oxygen atoms from DMF molecules. In contrast, the second Mn(II) ion is bonded to two nitrogen atoms from two [Ru(L^3^salen)(CN)_2_]^−^ units and four oxygen atoms contributed by four DMF molecules. Additionally, a different spatial arrangement of the cyanido bridges was observed for the hexacoordinated Mn(II) ions. The octahedral geometry of the Ru(III) center is defined by two imine nitrogen atoms and two phenoxide oxygen atoms from the Schiff base ion, as well as two cyanide ions. Ru–N_imine_ and Ru–O_phen_ bond lengths are observed within the ranges of 1.990–2.021 Å and 2.000–2.030 Å (Table 1) [24,31].

**Figure 30:**
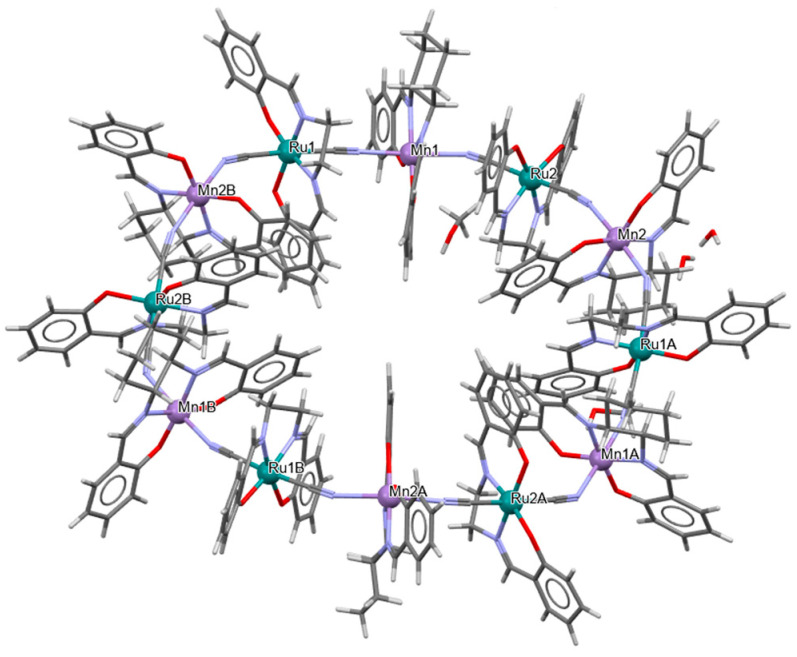
Molecular structure of **17a** VIVCOV [24,40].

The neutral dodecanuclear cluster containing a 36-membered macrocycle {[Ru^III^(L^8^salen)(CN)_2_][*R*,*R*-Mn^III^(L^1^salen)]}_6_·PPh_3_·6CH_3_CN·6MeOH·12H_2_O **17a** was synthesized in air by reacting a methanol solution of [PPh_4_][Ru(L^8^salen)(CN)_2_] with *R*,*R*-[Mn(L^8^salen)(H_2_O)_2_]ClO_4_ dissolved in a mixture of CH_3_OH/CH_3_CN [40]. In the crystal structure of **17a** (Figure 30, Table 1), both Ru(III) and Mn(III) ions are six-coordinate, each adopting a distorted octahedral geometry. The equatorial plane is defined by N_2_O_2_ donor atoms from the Schiff base ligand, while the axial positions are occupied by carbon or nitrogen atoms from the bridging cyanide ligands. In the case of the Mn(III) ion, the axial Mn–N_cyanide_ bond lengths are noticeably longer than the Mn–O_phen_ and Mn–N_imine_ distances, reflecting an elongated octahedral geometry characteristic of the Jahn–Teller effect (Table 1). Complex **17a** is very rare example of a cyanide-bridged 4*d*–3*d* metallic-based nano-sized chiral magnetic molecular wheel compound [40].

**Figure 31:**
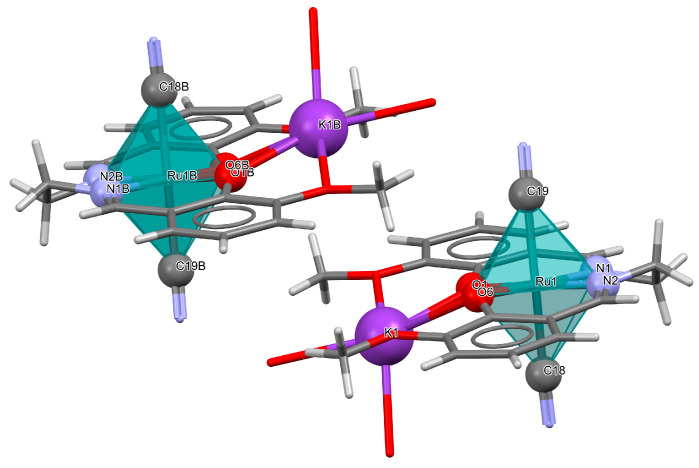
Molecular structure of **18a** YUFTAW [24,41].

The heteronuclear complex [K(H_2_O)_2_Ru^III^(L^8^salen)(CN)_2_]·H_2_O **18a** was synthesized via the reaction of [Ru^III^(L^8^salen)(PPh_3_)Cl] with KCN in methanol solution [41]. The Ru(III) ion (Figure 31, Table 1) is situated within the N_2_O_2_ donor set of the Schiff base ligand, with the two nitrogen and two oxygen atoms occupying the equatorial positions of its octahedral coordination environment, while the cyanide ligands are located at the axial sites. The Ru–N_imine_ bond lengths are measured at 1.982(9) and 2.000(9) Å, whereas the Ru–O_phen_ distances are 2.018(6) and 2.025(7) Å (Table 1). The K ion is situated in the open coordination pocket of the Schiff base ligand, where it is coordinated by two bridging phenoxo oxygen atoms, two methoxy oxygen atoms, and two water molecules. In the structure of **18a**, the presence of supramolecular dimeric units formulated as {[K^I^(H_2_O)_2_Ru^III^(L^8^salen)(CN)_2_]}_2_ is observed. These dimers are stabilized through weak non-covalent interactions, specifically anagostic contacts, formed between a hydrogen atom of a methyl group on one [K^I^Ru^III^] moiety and the potassium ion of a neighboring dimer. This interaction contributes to the overall stability and organization of the crystal structure, illustrating the role of subtle intermolecular forces in the formation of extended supramolecular architectures.

**Figure 32:**
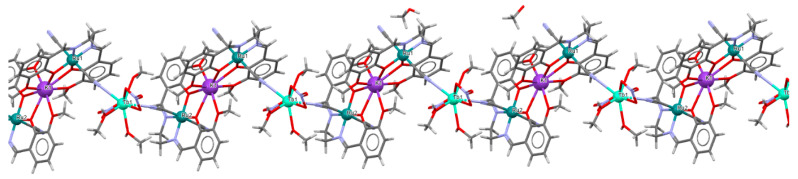
**Fragment of** one-dimensional chain of **18b** YUFTEA [24,41].

The isomorphous compound with a one-dimensional structure, ∞[{Ru^III^(L^8^salen)(CN)_2_KRu^III^(L^8^salen)(CN)_2_}{Ln(NO_3_)_2_(MeOH)_3_}]·2MeOH **18b** (Ln = Gd, Tb, Dy), was formed through the reaction between **18a** and respective lanthanide(III) nitrates [41]. In the crystal structure of **18b**, trinuclear cyanido-bridged units {Ru^III^−CN−Tb^III^−NC−Ru^III^} are linked via K ions, which are coordinated by two O_2_O_2_ donor sets from two distinct L^8^salen ligands, resulting in the formation of an infinite one-dimensional coordination polymer (Figure 32). Each [Ru^III^(L^8^salen)(CN)_2_]^−^ metalloligand coordinates to a Ln(III) ion via one bridging cyanide group, while the second cyanide ligand remains terminal. The Ln(III) center adopts a nine-coordinate geometry, bonded to two cyanide bridges, four oxygen atoms from two bidentate nitrate ligands, and three methanol molecules.

**Figure 33:**
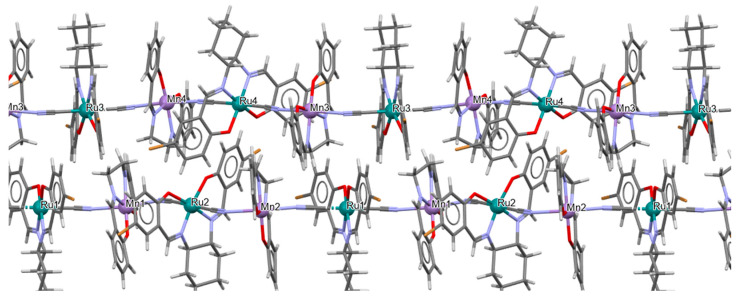
A view of an infinite one-dimensional zigzag chain of **19a** KILRAB [24,42]. The hanging contacts have been omitted for clarity.

**Figure 34:**
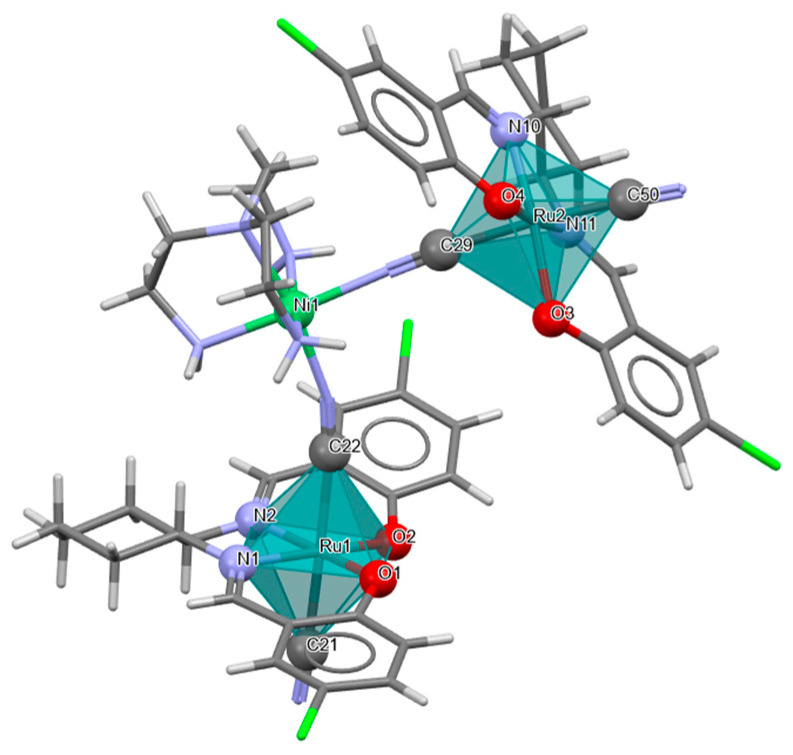
Molecular structure of **19c** KILRIJ [24,42].

Two pairs of chiral cyano-bridged heterobimetallic compounds, [Mn(L^8^salen)Ru^III^((*R*,*R*)*-*L^15^salen))(CN)_2_]_n_ **19a**, [Mn(L^8^salen)Ru^III^((*S*,*S*)*-*(L^15^salen))(CN)_2_]*_n_* **19b**, [Ni(tren)][Ru^III^((*R*,*R*)-(L^16^salen)(CN)_2_]_2_ **19c**, and [Ni(tren)][Ru^III^((*S*,*S*)-(L^16^salen))(CN)_2_]_2_ **19d** (where L^15^salen = dianion of *N*,*N*′-bis(5-bromosalicylidene)-*o*-cyclohexyldiamine, L^15^salen = dianion of *N*,*N*′-bis(5-chlorosalicylidene)-*o*-cyclohexyldiamine tren = tri(2-aminoethyl)amine) were obtained in MeOH/MeCN solution by the reaction of ((*R*,*R*) or (*S*,*S*))-[Ru^III^(L^15^salen/L^16^salen(CN)_2_]^−^ with [Mn(L^8^salen)(H_2_O)_2_]ClO_4_ or Ni(tren)(NO_3_)_2_, respectively [42]. The structures of an enantiomeric pair, **19a** and **19b** (Figure 33, Table 1), consist of a neutral cyano-bridged zigzag chain built from repeating (–Ru^III^–CN–Mn^III^–NC–)*_n_* units, with adjacent Mn(III) ions adopting a *trans* configuration. Each Ru(III) center adopts a slightly distorted octahedral geometry, coordinated by two nitrogen and two oxygen atoms from the Schiff base ligand, along with two carbon atoms from cyanide groups. The Mn(III) center adopts a highly distorted octahedral geometry, with the equatorial plane defined by two nitrogen and two oxygen atoms from the Schiff base ligand, while the axial sites are occupied by two nitrogen atoms from bridging cyanide ligands. The distances between Ru and N_imine_ range from 1.94 to 2.02 Å, while those between Ru and O_phen_ are 1.981–2.042 Å (Table 1). The axial bonds are noticeably longer than the equatorial ones, a consequence of the well-known Jahn–Teller distortion typically observed in high-spin Mn(III) ions with octahedral coordination. Compounds **19c** and **19d** (Figure 34, Table 1) are trinuclear complexes where the central Ni(II) ion is linked to two terminal (*R*,*R*/*S*,*S*)-*trans*-[Ru(L^16^salen)(CN)_2_]^−^ units via cyanide bridges. Each Ni(II) center exhibits a distorted octahedral coordination geometry, being six-coordinated, with four nitrogen atoms from the tren ligand and two nitrogen atoms from the cyanide groups of the two separate (*R*,*R*)-[Ru^III^(L^16^salen)(CN)_2_]^−^ units. Bond lengths between Ru(III) and N_imine_ are reported to be 1.97–2.01 Å, while those between Ru(III) and O_phen_ measure 1.976–2.016Å, as presented in Table 1. Intermolecular interactions connect each trinuclear molecule, leading to the formation of a two-dimensional (2D) supramolecular structure [42].

Schiff base ligands coordinate to ruthenium through imine nitrogen (Ru–N_imine_) and phenolic oxygen (Ru–O_phen_), forming chelate rings whose geometries are influenced by the oxidation state of Ru and the *cis*/*trans* configuration (Table 2) [25,26,27,28,29,30,31,32,33,34,35,36,37,38].

The Ru–O_phen_ bond lengths in Schiff base complexes are particularly sensitive to the oxidation state of ruthenium and the geometric arrangement of ligands. In general, higher oxidation states of Ru, with some exceptions, result in shorter Ru–O_phen_ bonds due to increased electrostatic attraction between the positively charged metal center and donor atoms of the Schiff base ligands. The Ru–N_imine_ bond lengths are less affected by changes in the oxidation state of ruthenium. The isomeric form of the complex also influences the lengths of both the Ru–O_phen_ and Ru–N_imine_ bonds. The *trans* geometry generally leads to shorter Ru–O_phen_ bond lengths than the *cis* form, likely due to reduced steric repulsion and differences in electronic delocalization. In *cis* isomers, the metal–ligand interactions are more sterically hindered in the coordination environment, resulting in longer Ru–O_phen_ bond lengths. Isomerism has a more subtle effect on Ru–N_imine_ bond lengths than on Ru–O_phen_ bonds. The *cis* isomers typically exhibit slightly longer and more variable Ru–N_imine_ distances, likely due to steric interactions between ligands and uneven electron distribution. In contrast, *trans* isomers maintain shorter and more consistent Ru–N_imine_ bond lengths, reflecting a more symmetrical coordination environment and reduced steric hindrance.

## 3. The Potential Applications of Ru(II/III/VI)–Salen Complexes

**Figure 35:**
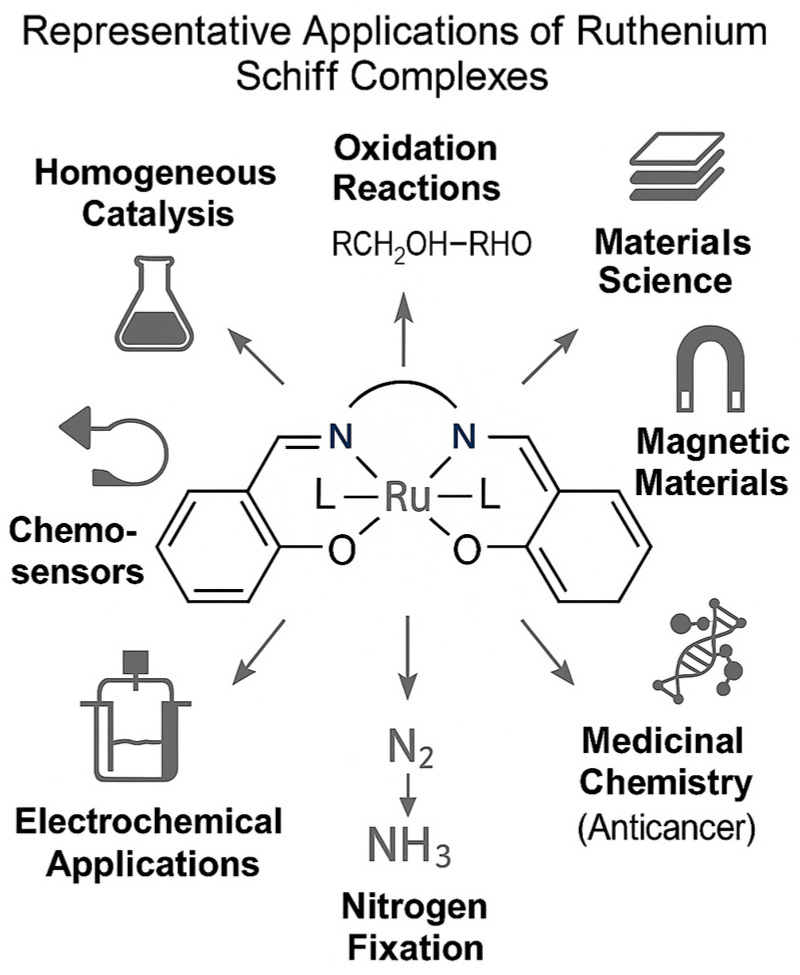
Potential applications of ruthenium(II/III/VI)–salen complexes.

Ru(II/III/VI)–salen complexes represent a fascinating class of coordination compounds that have attracted increasing interest due to their structural tunability, redox versatility, and stability under diverse conditions. The potential applications of these materials have been explored across a range of domains, including catalysis, medicine, and advanced materials science (Figure 35).

**Figure 36:**
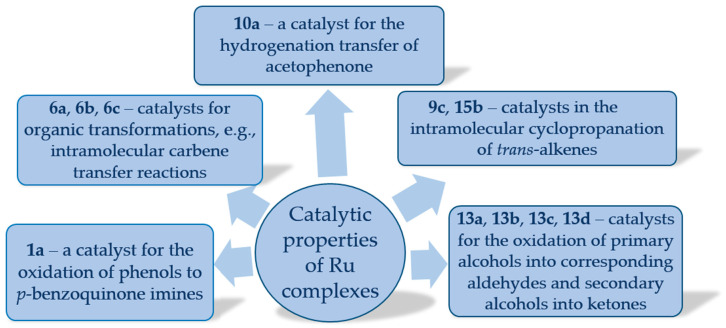
Potential catalytic applications of Ru–salen complexes.

**Figure 37:**
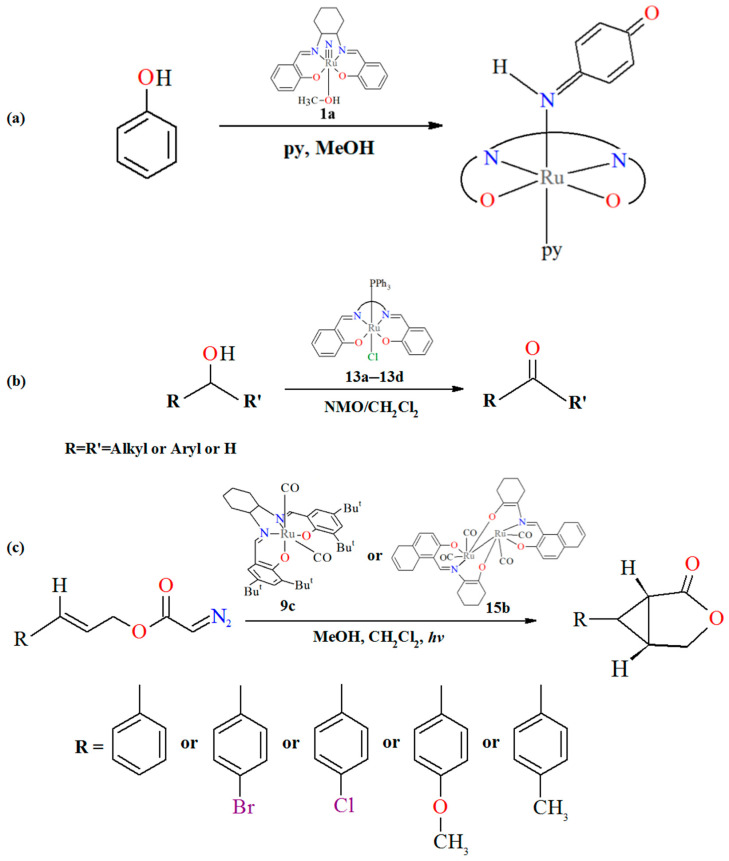
(**a**) Scheme of the oxidation of phenols to *p*-benzoquinone imines by Ru–salen complexes. (**b**) Scheme of catalytic oxidation reactions of alcohols to aldehydes/ketones catalyzed by Ru–salen complexes. (**c**) Scheme of the intramolecular cyclopropanation of allylic diazoactates catalyzed by Ru–salen complexes.

In catalysis, for example, they can act as efficient and selective mediators in oxidation and hydrogenation reactions (Figure 36 and Figure 37). In materials science, their redox versatility and structural tunability facilitate the design of functional materials, including molecular switches and conductive polymers. In medicinal chemistry, their capacity to interact with biomolecules and regulate redox processes is driving ongoing efforts toward the development of anticancer, antimicrobial, and diagnostic agents.

Ruthenium–salen complexes with *trans* configurations are investigated as catalysts in a wide variety of organic transformations. Their stability, predictable geometry, and well-defined electronic structures have made them attractive model systems for mechanistic studies as well as practical applications, e.g., the nitrido Ru(VI) complex **1a** exhibits rich chemical reactivity, as demonstrated by its ability to oxidize phenols to *p*-benzoquinone imines. The proton-coupled electron-transfer reactions of phenols have attracted a great deal of attention due to their fundamental importance and relevance to numerous biological processes. In the case of **1a**, the reaction proceeds through a two-step sequential mechanism. Initially, an electrophilic attack by **1a** on the aromatic ring generates a Ru(IV) *p*-hydroxyanilido intermediate. In the subsequent step, a pyridyne-assisted intramolecular redox process takes place, resulting in the formation of the Ru(II) *p*-benzoquinone imine product. This transformation highlights the strong oxidative potential of the metal center and the important role of the Schiff base ligand in stabilizing high-valent ruthenium species, thereby enabling selective activation of phenolic O–H bonds [7,25]. An investigation into the catalytic properties of **10a** revealed that it is an effective catalytic precursor for the hydrogenation transfer of acetophenone [34]. Complexes **13a**, **13b**, **13c**, and **13d** have been shown to effectively catalyze the oxidation of primary alcohols into their corresponding aldehydes and secondary alcohols into ketones, using dichloromethane as the solvent and N-methylmorpholine-N-oxide (NMO) as a co-oxidant [37].

The *cis*-Ru–salen complexes, though less studied than their *trans* counterparts, exhibit unique reactivity and, in some cases, superior catalytic performance. The nonequivalent coordination sites in the *cis* configuration create distinct steric and electronic environments, offering a powerful platform for the design of asymmetric catalysts. These features position *cis*-Ru–salen complexes as promising candidates for enantioselective synthesis and novel catalytic applications, e.g., complexes **6a**, **6b**, and **6c** are reported as catalysts for organic transformations, including intramolecular carbene transfer reactions [30], the mononuclear **9c** and dinuclear ruthenium **15b** complexes exhibit high catalytic efficiency in the intramolecular cyclopropanation of *trans*-allylic diazoacetates under irradiation with a 300 W incandescent lamp in the absence of light exposure (the irradiation can enhance the dissociation rate of CO ligands from carbene M(CO)_2_-type complexes to give more reactive mono(carbonyl) species). The corresponding cyclopropanation products were obtained in yields ranging from 84% to 96% (**9c**) and 85% to 94% (**15b**), respectively [22,33,39]. In the case of **15b**, the influence of solvent on this transformation was examined, revealing that coordinating solvents such as MeOH or THF suppressed the reaction, whereas the use of the non-coordinating solvent CH_2_Cl_2_ containing 0.05% (v/v) MeOH afforded superior results [39].

The cytotoxicity profiles of ruthenium(III) complexes **3a** and **3b** were evaluated using several human cancer cell lines, including HeLa (cervical), A549 (lung), MCF-7 (breast), and HepG2 (liver), which are commonly employed in the biological assessment of metal-based drugs. It is important to highlight that the anticancer mechanisms of these complexes are strongly influenced by the nature of the axial ligand (guanidine or amidine). Remarkably, their modes of action differ substantially from those of the clinically established drug cisplatin [27].

Converting metal nitrides [Ru^VI^(L^1^salen)(N)(OH_2_)]^+^ into ammonia **4a** represents a crucial step in both biological and chemical nitrogen fixation. The straightforward reduction of a Ru(VI)–nitrido complex containing a Schiff base ligand to a Ru(III)-ammonia species by L-cysteine in aqueous solution constitutes a rare example of metal nitrido reduction. This transformation provides valuable insight into the redox behavior of nitrido complexes under aqueous conditions [28].

The synthetic strategy employed to prepare complexes **5a** and **5b** provides an innovative and effective approach to the production of arylamidine-containing metal complexes. Amidines are a fundamental class of functional group in both medicinal and coordination chemistry. Their well-documented roles range from acting as pharmacophores in drug discovery to serving as highly adaptable ligands that can fine-tune the steric and electronic environment of metal centers [29]. Therefore, developing new methodologies to access arylamidine derivatives is of significant interest in order to expand the toolbox of both bioinorganic and organometallic chemistry.

Compound **7a** is a key precursor in the synthesis of novel, cyanide-bridged, heterometallic coordination polymers of the {Ru(III)–Mn(II)/Co(II)/Ni(II)} type [31]. These coordination polymers are of great interest due to their adjustable architectures and multifunctional properties, which result from the interaction between the ruthenium(III) centers and the incorporated 3*d* transition metals. These materials have potential applications in areas such as molecular magnetism, electronic and photonic devices, and catalytic processes. This highlights the importance of developing efficient synthetic methods for preparing them.

The novel hybrid material **8a**, when immobilized on SBA-15, has been shown to exhibit significant antibacterial and anti-biofilm activity against the bacteria *Enterococcus faecalis*, *Staphylococcus aureus*, *Pseudomonas aeruginosa*, and *Escherichia coli*. Furthermore, in vitro assays assessing its cytotoxic effects on HeLa cells demonstrated a dose-dependent reduction in cell viability, highlighting the compound’s considerable potential for biomedical applications [32].

Compound **11a** was investigated as a potential chemosensor for the selective recognition of acetate anions, which play a crucial role in various metabolic processes, such as enzyme activity and antibody function, and can form strong hydrogen bonds with different donor groups, including urea and boronic acid derivatives. The results showed that the fluorescence intensity of **11a** increased with rising acetate concentrations, with no interference from other anions [35].

Complexes **14a** and **14b**, incorporating planar N_2_O_2_ ligands with π-extended aromatic systems, efficiently release nitric oxide (NO) under visible-light irradiation, highlighting their potential as photoresponsive NO donors [38].

Magnetic studies of the cyanido-bridged heterometallic coordination polymers **16a, 16b** and **16c** revealed antiferromagnetic interactions between Ru(III) and either Mn(II) or Co(II) ions. In contrast, ferromagnetic coupling was observed between Ru(III) and Ni(II) centers. These findings emphasize the pivotal role of the cyanide ion, whose strong bridging ability and effectiveness in facilitating exchange interactions establish it as a vital linker in the formation of heterometallic structures. Cyanide-bridged compounds can generate systems with tunable spin arrangements by enabling diverse magnetic coupling pathways, thereby offering significant potential for the development of molecule-based magnetic materials and multifunctional coordination polymers [31]. The subsequent compound, **17a**, a cyanide-bridged 4d–3d heterobimetallic chiral macrocyclic enantiomeric complex, emerges as a particularly promising candidate for advanced magnetic material applications. In-depth magnetic investigations have demonstrated that **17a** exhibits pronounced single-molecule magnet (SMM) behavior, characterized by slow magnetization relaxation and magnetic bistability at the molecular level [40].

The chiral cyano-bridged bimetallic systems **19a**, **19b**, **19c**, and **19d** are also promising candidates for the design of magnetic materials, such as molecular multiferroics and chiral magnets, owing to their delocalized magnetic orbitals, pronounced magnetic anisotropy driven by strong spin–orbit coupling, and capacity to adopt multiple oxidation states with varying coordination environments. Magnetic investigations revealed antiferromagnetic interactions between the Ru(III) and Mn(III) centers bridged by cyanide ligands (**19a**, **19b**), while ferromagnetic coupling occurs between Ru(III) and Ni(II) ions (*J* = 2.73 cm^−1^) (**19c**) [42].

## 4. Conclusions

Ruthenium(II/III/VI)–salen complexes can be obtained using various synthetic methods. These include the direct coordination of pre-formed salen ligands to ruthenium precursors; stepwise assembly from salicylaldehyde and amine components in the presence of a ruthenium salt; and post-synthetic modification of pre-assembled ruthenium–ligand frameworks. The oxidation state of the metal center and the geometry of the complex can be controlled by tuning reaction conditions such as solvent, temperature, counterion, and oxidizing or reducing agents. The modular nature of the salen ligand framework not only stabilizes the metal center but also allows for systematic modification of the steric and electronic environment around the metal center. The complexes usually adopt an octahedral *trans* configuration, with the ruthenium(II/III/VI) center being tightly bound to two nitrogen and two oxygen atoms of the tetradentate Schiff base ligands. In addition, neutral (e.g., CO, NH_3_, CH_3_OH, CH_3_CN) or anionic (e.g., Cl^−^) ligands occupy the two vacant sites. In the case of cationic complexes, counterions such as ClO_4_^−^ or PF_6_^−^ are present in their crystal structure. Ru(II/III/VI)–salen complexes have potential applications in many fields, including catalysis, materials science, and medicinal chemistry. Their stability and redox activity enable their use in the design of advanced functional materials, sensors, and molecular devices. Furthermore, due to their ability to interact with biological molecules and generate reactive oxygen species, Ru–salen complexes are being investigated for applications in anticancer therapy and photodynamic treatments.

Future Perspectives: The multifunctionality of ruthenium–salen complexes highlights their potential to act as molecular platforms for interdisciplinary applications. Advances in ligand design, computational modeling, and experimental techniques are expected to enable these complexes to continue providing innovative solutions in catalysis, materials science, and biomedicine. Future research could focus on designing Ru(II/III/VI) complexes that are multifunctional and have enhanced selectivity in C–H, C–O, C–N, and C–C bond activation. These complexes would also have tunable redox properties for sustainable oxidation reactions. Additionally, light-responsive ruthenium–salen complexes containing a Ru–NO or Ru–CO bond offer potential for use in photochemical and theranostic applications, combining catalysis with the controlled release of reactive species or drug molecules. In summary, their ability to combine catalytic, optical, biological, and electrochemical properties within a single molecular structure establishes them as one of the most promising and versatile classes of coordination compounds.

## Figures and Tables

**Table 1:** The formula and selected bond distances of Ru(II)–, Ru(III)–, and Ru(VI)–salen complexes [24].

The Complex Formula	CSD Refcodes	Ru–N_imine_ (Å)	Ru–O_phen_ (Å)	Ref.
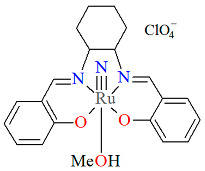 **1a**	IQIBUF	2.030(3) 2.018(4)	1.977(3) 1.971(3)	[25]
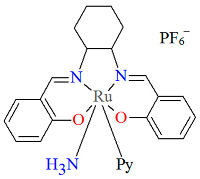 **2a**	EVEHAQ	1.994(3) 1.988(2)	2.015(2) 2.021(2)	[26]
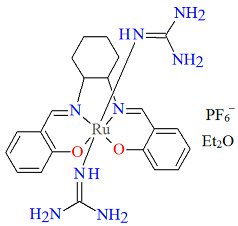 **3a**	LEJLIY	1.984(4) 1.990(4)	2.030(3) 2.023(2)	[27]
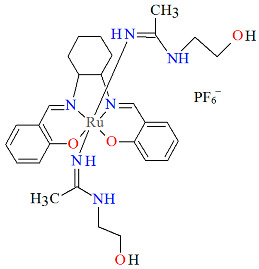 **3b**	LEJLOE	1.981 1.981	2.032 2.032	[27]
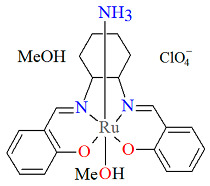 **4a**	NIGDEP	1.989(3) 1.988(3)	2.015(2) 2.028(2)	[28]
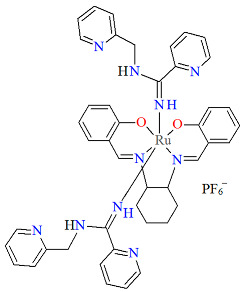 **5a**	QAKJIZ	1.987(2) 1.989(3)	2.024(2) 2.015(2)	[29]
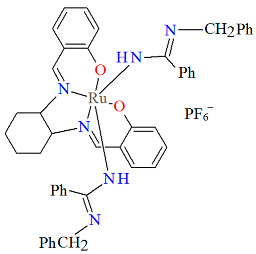 **5b**	QAKJOF	1.990(3) 2.067(3)	2.023(2) 2.027(2)	[29]
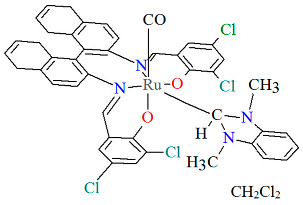 **6a**	ERUQIU	2.123(4) 2.054(4)	2.060(4) 2.089(3)	[30]
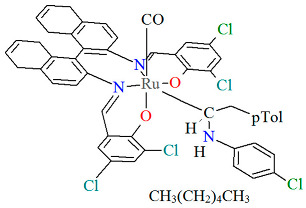 **6b**	ERUQOA	2.163(3) 2.036(3)	2.078(2) 2.101(2)	[30]
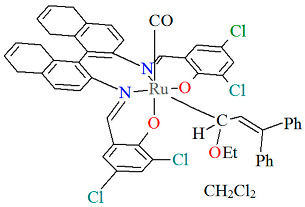 **6c**	ERUQUG	2.055(2) 2.178(2)	2.070(2) 2.082(2)	[30]
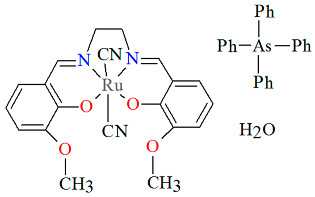 **7a**	GOJKEY	1.980(7) 1.975(7)	2.025(6) 2.004(4)	[31]
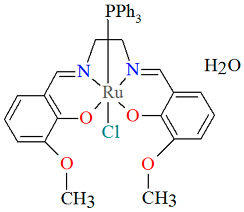 **8a**	HUKLEH	1.958(8) 1.985(9)	2.008(6) 2.009(7)	[32]
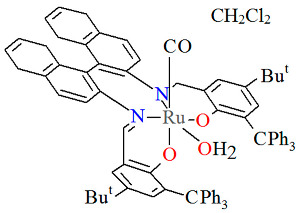 **9a**	KUSVAY	2.026(3) 2.052(4)	2.081(4) 2.071(3)	[33]
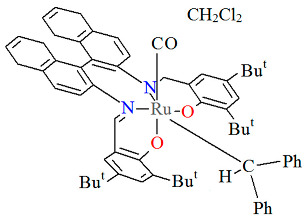 **9b**	KUSVEC	2.031(3) 2.202(3)	2.064(2) 2.060(2)	[33]
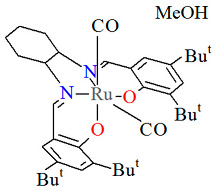 **9c**	KUSXII	2.073(2) 2.052(2)	2.060(1) 2.101(1)	[33]
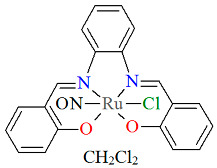 **10a**	LEQBAN	2.020(4) 2.022(4)	2.025(3) 2.023(4)	[34]
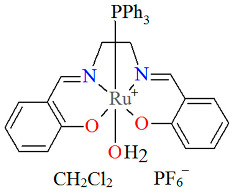 **11a**	MEKGIV	2.003(6) 1.983(6)	2.008(5) 2.007(6)	[35]
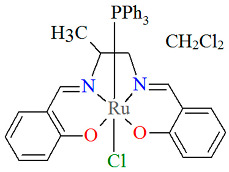 **12a**	YOBXAR	1.983(3) 2.008(3)	2.016(3) 2.008(3)	[36]
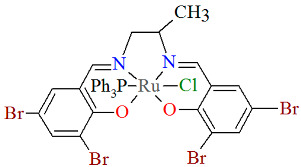 **12b**	YOBXEV	1.981(6) 1.966(7)	2.102(5) 2.105(5)	[36]
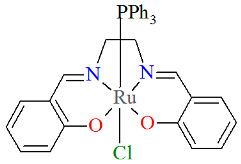 **13a**	ZIBNOQ	1.987(2) 1.992(2)	2.011(2) 2.022(2)	[37]
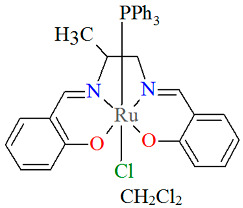 **13b**	ZIBNUW	2.009(5) 1.986(5)	2.016(4) 2.014(4)	[37]
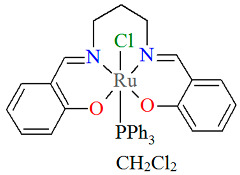 **13c**	ZIBPAE	2.035(4) 2.051(5)	2.007(4) 2.014(3)	[37]
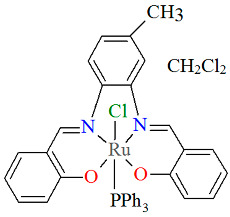 **13d**	ZIBPEI	2.017(4) 2.005(4)	2.004(3) 2.012(3)	[37]
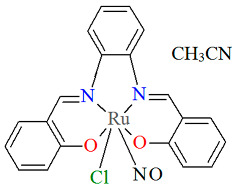 **14a**	CENXAY	2.031(5) 2.028(5)	2.037(4) 2.035(4)	[38]
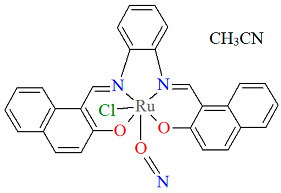 **14b**	CENXIG	2.005(3) 2.007(2)	2.027(2) 2.033(3)	[38]
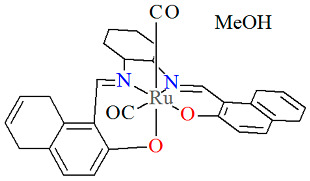 **15a**	ECEZIZ	2.065(2) 2.038(2)	2.092(1) 2.041(1)	[39]
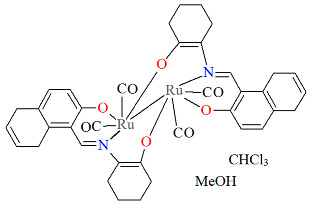 **15b**	ECEZOF	2.066(3) 2.075(4)	2.078(3) 2.076(3) 2.092(3) 2.218(4) 2.066(3)	[39]
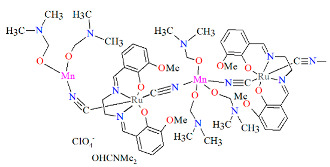 **16a**	GOJHAR	2.004(6) 1.992(4) 1.996(4) 2.008(4)	2.026(4) 2.016(3) 2.019(4) 2.024(4)	[31]
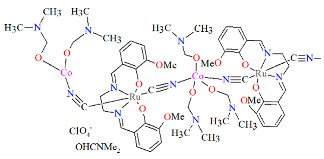 **16b**	GOJGUK	1.999(4) 2.021(6) 1.993(5) 1.999(5)	2.026(3) 2.029(5) 2.028(4) 2.030(4)	[31]
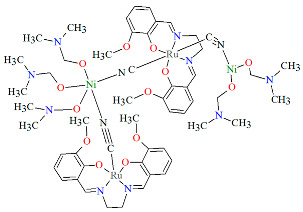 **16c**	GOJKAU	1.965(7) 1.993(9) 1.993(8) 1.990(8)	2.025(6) 2.000(5) 2.004(5) 2.022(7)	[31]
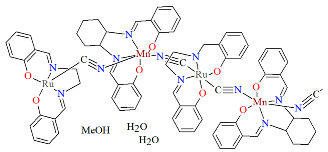 **17a**	VIVCOV	1.99(3) 1.94(2) 2.03(2) 2.01(2)	2.03(1) 1.95(2) 2.06(1) 2.01(1)	[40]
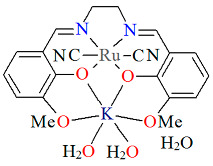 **18a**	YUFTAW	1.982(9) 2.000(9)	2.018(6) 2.025(7)	[41]
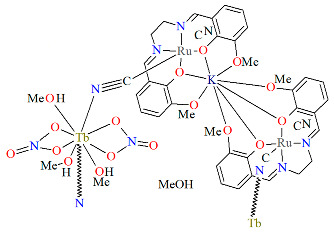 **18b**	YUFTEA	1.971(7) 1.967(9) 1.992(6) 1.994(8)	2.019(7) 2.008(6) 2.016(6) 2.009(5)	[41]
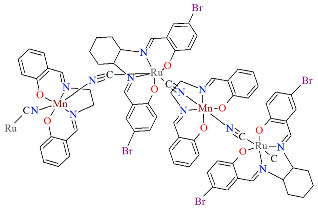 **19a**	KILRAB	1.94(1) 1.983(7) 2.02(1) 1.980(8)	1.988(8) 1.981(6) 1.987(6) 2.042(7)	[42]
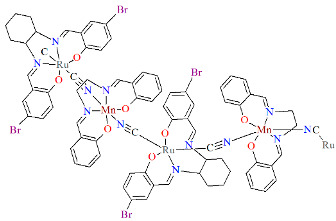 **19b**	KILREF	2.00(1) 1.997(9) 2.01(1) 2.002(7)	2.009(6) 1.994(9) 2.017(6) 2.010(9)	[42]
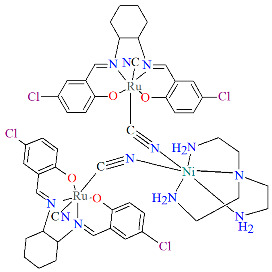 **19c**	KILRIJ	2.01(1) 1.97(1) 1.99(1) 1.97(1)	1.976(9) 2.002(7) 2.016(8) 1.980(9)	[42]
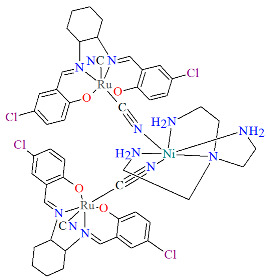 **19d**	KILROP	2.008(4) 1.996(4) 1.984(5) 1.987(5)	1.990(4) 2.006(4) 1.995(4) 1.989(4)	[42]

**Table 2:** Average Ru–O_phen_ and Ru–N_imine_ bond lengths in salen–ruthenium complexes depending on oxidation state and *cis*/*trans* isomerism [25,26,27,28,29,30,31,32,33,34,35,36,37,38,39].

Ru Oxidation State	Geometry	Bond Length (Å)		Ref.
		Ru–O_phen_	Ru–N_imine_	
II	*cis*	2.06–2.10	2.03–2.20	[30,33,39]
II	*trans*	2.02–2.05	2.02	[34]
III	*trans*	2.00–2.11	1.96–2.07	[26,27,28,29,31,32,35,36,37,38]
VI	*trans*	1.97–1.98	2.02–2.03	[25]

## Data Availability

No new data were created or analyzed in this study.
